# BCCTBbp: the Breast Cancer Campaign Tissue Bank bioinformatics portal

**DOI:** 10.1093/nar/gku984

**Published:** 2014-10-20

**Authors:** Rosalind J. Cutts, José Afonso Guerra-Assunção, Emanuela Gadaleta, Abu Z. Dayem Ullah, Claude Chelala

**Affiliations:** Centre for Molecular Oncology, Barts Cancer Institute, Queen Mary University of London, Charterhouse Square, London EC1M 6BQ, UK

## Abstract

BCCTBbp (http://bioinformatics.breastcancertissue bank.org) was initially developed as the data-mining portal of the Breast Cancer Campaign Tissue Bank (BCCTB), a vital resource of breast cancer tissue for researchers to support and promote cutting-edge research. BCCTBbp is dedicated to maximising research on patient tissues by initially storing genomics, methylomics, transcriptomics, proteomics and microRNA data that has been mined from the literature and linking to pathways and mechanisms involved in breast cancer. Currently, the portal holds 146 datasets comprising over 227 795 expression/genomic measurements from various breast tissues (e.g. normal, malignant or benign lesions), cell lines and body fluids. BCCTBbp can be used to build on breast cancer knowledge and maximise the value of existing research. By recording a large number of annotations on samples and studies, and linking to other databases, such as NCBI, Ensembl and Reactome, a wide variety of different investigations can be carried out. Additionally, BCCTBbp has a dedicated analytical layer allowing researchers to further analyse stored datasets. A future important role for BCCTBbp is to make available all data generated on BCCTB tissues thus building a valuable resource of information on the tissues in BCCTB that will save repetition of experiments and expand scientific knowledge.

## INTRODUCTION

Breast carcinoma is the leading cause of cancer-related death among women, accounting for 23% of total cancer cases and 14% of cancer deaths (1). It is a heterogeneous disease presenting a variety of morphological features as well as a range of genetic, epigenetic and transcriptomic alterations (2).

Advances in research have resulted in a huge improvement in the outcome for patients. However, breast cancer still remains a major public health issue with only three robust biomarkers predicting its response to endocrine (oestrogen receptor and progesterone receptor) or biological therapies (human epidermal growth factor receptor 2 (HER2)). Despite the definite benefit of administering hormonally-targeted treatments, we still do not know why some patients respond to these therapies while others either do not respond or develop resistance, which has serious clinical implications.

A wide scale review involving over 50 of the world's leading breast cancer researchers highlighted the inaccessibility of tissue samples and materials as a major obstacle in translating science into new treatments, with researchers sometimes spending months tracking down suitable samples with well annotated clinical data (3). To fill this void and create a vital resource of breast cancer tissue for researchers across the UK and Ireland, the Breast Cancer Campaign Tissue Bank (BCCTB) was established in 2010 as a unique collaboration between leading UK research institutions (http://www.breastcancertissuebank.org) (4).

Associated with the Tissue Bank is a powerful bioinformatics portal (BCCTBbp) dedicated to maximising research on patient tissues. The BCCTBbp was developed using BioMart, a well established, freely available bioinformatics data management framework (5) to provide mutualistic support to the Tissue Bank. The BCCTBbp functions as an online literature-mining resource, permitting users to examine and collate published breast cancer data from a single portal. BCCTBbp currently stores data that has been mined from the literature on genes, transcripts, proteins, non-coding RNA and copy number events and links this to pathways and mechanisms involved in breast cancer. It has an integrated analytical layer to enable researchers not only to mine but also to analyse breast cancer literature data from multiple sources.

Seamless interoperability between the Tissue Bank and the bioinformatics portal allows researchers to examine published findings prior to applying for tissues. This complementarity between the clinical data and samples available from the Tissue Bank and the literature-derived experiments provided by the BCCTBbp exemplify how biology and bioinformatics can be used synergistically to maximise research output. Also, in future the BCCTBbp will store experimental results generated on BCCTB tissues to make these available to the whole community. Each BCCTB sample will be linked to all its associated experimental findings which will ensure that researchers will have access to maximal data for every sample of interest, thus optimising data sharing opportunities while minimising duplication of effort. While already undoubtedly an invaluable resource, the full potential of the symbiotic relationship between the Tissue Bank and bioinformatics portal will become reality as more experimental data from BCCTB-provided samples is returned and stored alongside the corresponding clinical information.

Here we describe its current data content, query system and analytical capabilities.

## CONSTRUCTION AND DATA CONTENT

The aim of the portal is to store, mine and integrate diverse breast cancer datasets to produce a comprehensive resource for breast cancer scientists. In order to achieve this, we designed a robust internal structure encompassing the details of breast cancer publications, experimental setup, detailed clinical annotations and also experimental findings. Inherent to the portal is a BioMart-compatible MySQL database that stores this information in two datasets:

- ‘Study Dataset’ contains information about the published articles and details on the patients/clinical samples used in each experiment/publication.
- ‘Gene Dataset’ collates data on the genes, transcripts, proteins, probesets, miRNAs and other biological features reported to be expressed/regulated/altered in each specific experiment. The portal currently supports expression, proteomics, miRNA, genomics and methylation experimental data types, although the flexibility of the portal design enables other experimental data types to be easily added as required.

The articles to be included in the database are selected by periodic PubMed searches as well as suggestions from breast cancer scientists and peers. Data from the original articles are then manually curated, reviewed for accuracy and consistency and finally loaded into the database. An extensive controlled vocabulary is employed to record specific details on the clinical samples used in each study (Supplementary Table S1). This includes detailed information on the provenance of samples including extraction and preparation methods; cellular components; sample type; malignant and benign lesions; molecular subtypes; immunochemistry markers as well as chemotherapy and drug treatments (where available). Gene identifiers are extracted from each relevant publication and mapped to the Ensembl human gene annotation system (version 69). Genomic coordinates are standardised to GRCh37/hg19 using the liftOver tool from UCSC (6).

Currently, BCCTBbp contains manually curated breast cancer data extracted from 83 published ‘omics’ studies comprising 146 datasets of which there are 104 transcriptomics, 8 proteomics, 5 methylation, 11 miRNA and 18 genomics datasets. The database contains 227 795 expression/genomic measurements on 14 742 genes, 91 119 transcripts and 112 microRNAs in over 16 000 samples representing various normal, benign and malignant tissues, body fluids, cell lines and murine models under different treatment conditions.

The BCCTBbp gene information is supplemented with key annotations from Ensembl including genes, transcripts, proteins, SNPs, genomic features, gene ontologies and multi-species data. This expands the variety of queries that can be performed and by utilisation of standard identifiers enables joint queries with other relevant biological datasets using BioMart data federation technology. To assess the quality of data extracted from publications and also to provide a uniform view of findings, selected datasets have been reanalysed using an in-house data analysis pipeline (7). Obtained results have been included in BCCTBbp and are made available through a dedicated analytical interface allowing users to interrogate these datasets and run further explorations.

## DATA ACCESS

BCCTBbp provides a number of access routes to accommodate different user types from basic querying to more complex data mining capabilities.

### Advanced/BioMart query

The most comprehensive query mode is available through an integrated MartView web interface. This provides a customised web interface with a multitude of querying options based on the extensive clinical and experimental parameters represented in the database. It follows the conventional BioMart set up of Filters and Attributes that will be familiar to any user of the numerous BioMart resources available. A simple query involves selecting one of the two BCCTBbp datasets available, setting up Filters to restrict searches to specific criteria, and choosing the Attributes to be included in the results. Below we outline the two available datasets for query with related Filters and Attributes.

(i) The BCCTB Bioinformatics Gene Data provides a number of modules for querying and extracting data by experiment/technology, publication/comparison as well as numerous options based on the study/sample controlled vocabulary. Further modules for transcriptomics, proteomics, genomics and miRNA profiling are available and allow users to seamlessly link datasets based on the genes reported in an experiment. This provides a powerful way to build up large queries to answer complex scientific questions. Additional filter options are included from external data sources including KEGG pathways as well as gene information, chromosomal regions, gene ontology terms, expression, protein domains, multispecies queries and variations based on Ensembl data to provide a diverse range of queries and export options. The user has the choice to add multiple attributes from the module categories to the data before the results are either displayed via the web interface or exported in csv, excel or other formats. A further BioMart feature is the ability to merge this output data with other datasets such as UniProt, Reactome and other relevant datasets through the BioMart central portal (www.biomart.org).

**Figure 1.**
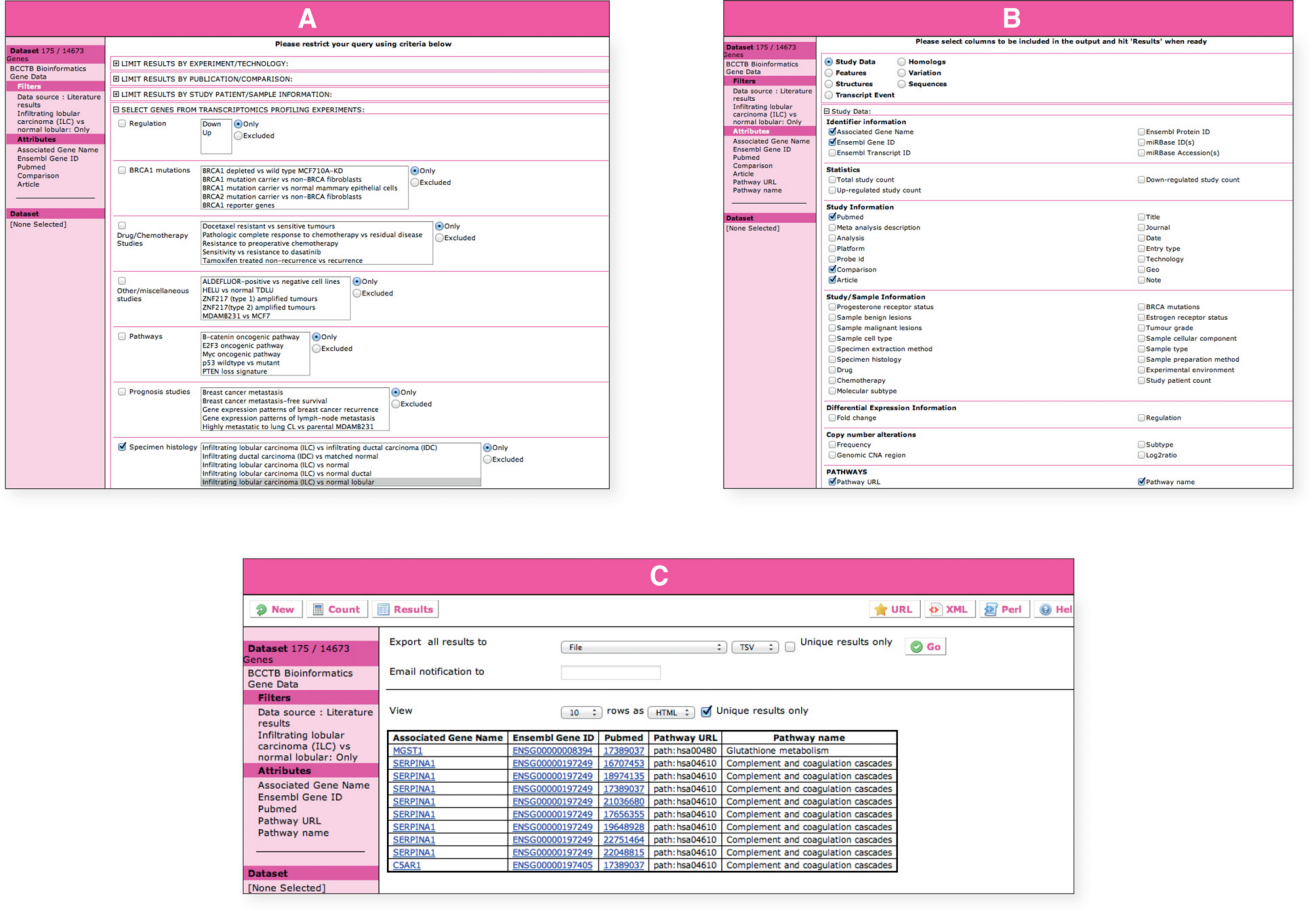
Accessing the BCCTBbp data through the MartView web query interface. In this first example our goal was to identify differentially regulated genes in Infiltrating Lobular Carcinoma (ILC), related pathways and check if any of these genes appear in the Parker PAM 50 gene set. The MartView interface can be accessed by following the ‘Advanced/BioMart Search’ button on the main bioinformatics portal web page (http://bioinformatics.breastcancertissuebank.org). The query starts by choosing the ‘BCCTB Bioinformatics Portal’ from the ‘Choose Database’ drop-down selection in the right panel. Users will be automatically directed to choose a dataset from the ‘Choose Dataset’ drop-down menu. (**A**) The ‘BCCTB Bioinformatics Gene Data’ dataset can be chosen from the dropdown menus. The left panel will refresh automatically displaying the ‘Filters’ and ‘Attributes’ nodes with their default settings. The next step involves choosing the appropriate attributes and filters to restrict the query. Clicking on the ‘Filters’ or ‘Attributes’ nodes on the left will display the related page on the right where ‘Filters’ or ‘Attributes’ are arranged into sections, which can be expanded/collapsed using the ‘+/−’ box. To choose an attribute or a filter, users can simply click on the checkbox next to its description. A summary of the selected filters and attributes is automatically displayed in the left panel. We will click on Filters in the left panel and then expand the ‘SELECT GENES FROM TRANSCRIPTOMICS PROFILING EXPERIMENTS:’ section. From the rich list of possible options, we will select ‘Specimen histology’ then ‘ILC versus normal lobular’ from the related list. Clicking on the ‘Count’ button in the tool bar at any time when constructing the query will return the number of genes satisfying the pre-selected criteria. (**B**) Clicking on the Attributes tab in the left panel allows the user to select which attributes of the data will be returned in the results. On the right panel, these are arranged into six modules for ‘Study Data’, ‘Features’, ‘Structures’, ‘Transcript Event’, ‘Homologs’, ‘Variation’ and ‘Sequences’. In order to select the output content, the ‘Attributes’ node on the left needs to be clicked on and the attribute page on the right needs to be chosen. In this example we are interested in exploring the de-regulated pathways. From the ‘Study Data’ attribute page, a selection is made of Pathway URL and Pathway name options. Finally, pressing the results button on the top left of the page will produce a table containing the requested information (**C**). One can select the option to download the results to a file at the top of the result page and export them using the ‘GO’ button. Again, there are options to change the format (‘HTML’, ‘CSV’ for comma separated values, ‘TSV’ for tab separated values, ‘XLS’ for Excel, ‘ADF’ for array description format) and whether to make the results unique. One can select a compressed file output and the query will run in the background to be downloaded later. One needs to provide an email address to receive an URL in a notification email that allows the query results to be downloaded. There is an option to produce a Perl script to repeat the query programmatically with BioMart Perl API. Due to the nature of the database and the one-to-many relationship between genes and transcripts, some results may appear more than once. To address that there is a button that can be used to present only unique results and the transcript attribute could be removed from the final results.

A typical query is shown in Figure 1 where we can investigate differences in specimen histological types using the transcriptomics profiling module. Here we investigate the differences between Invasive Lobular Carcinoma (ILC) and normal lobular cells and obtain a list of 175 transcripts of significance and related pathways (Figure 1). The portal allows multiple further filtering to enhance this initial query if required. For example, we can intersect this output to see if any of these genes appear in the Parker PAM 50 gene set (17) and extract related upstream sequences (Supplementary Figure S1).

Supplementary Figure S2 shows how it is possible to combine data from genomics and transcriptomics experiments and perform more complex queries. Here we look for potential genes of importance in basal breast cancer subtypes. The flexibility of BioMart then allows the output to be combined with different data types such as gene information, study data and/or relevant pathways.

(ii) The BCCTB Bionformatics Study Data provides a quick look up of clinical data from all studies in the database. Data could be queried by patient/sample details (such as related histology, cellular component, metastasis site and treatment) and/or patient/sample characteristics (such as menopause status, receptor status, gender and grade). This dataset is also integrated with the gene dataset. Links to the BCCTB Sample Finder are achieved through this layer.

### Quick search

We also provide a quick search for users to mine publications in the database where their particular gene of interest is reported using standard gene symbols. The quick search box has an autocomplete function. The results contain a summary of the studies reporting a link between the search term and breast cancer. This allows users to quickly validate or check the current knowledge about the role of a gene/protein in breast cancer.

### Association with the BCC Tissue Bank Sample Finder

**Figure 2.**
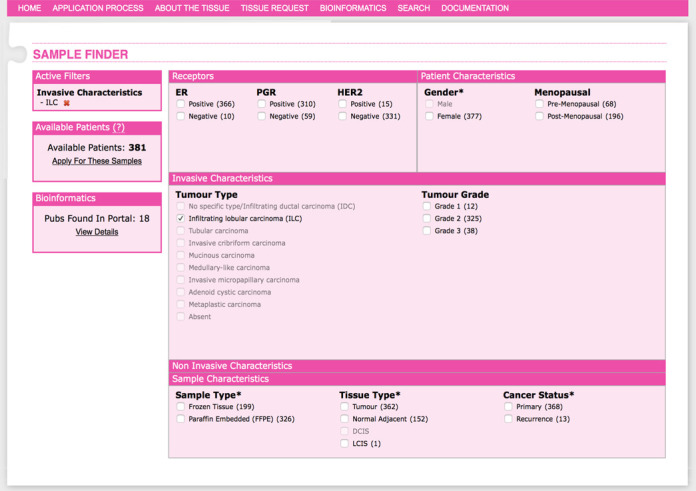
Accessing the BCCTBbp data through the BCCTB Sample Finder. The BCCTB sample finder (https://breastcancertissuebank.org/bcc/tissueBank?Name=sampleFinder, described elsewhere, manuscript in peer-review) is used to find samples within the Tissue Bank matching characteristics of interest. Here the Sample Finder is used to retrieve ILC samples available from BCCTB. On the lower left corner, the bioinformatics section indicates 18 studies stored in the BCCTBbp with ILC samples. Clicking on the hyperlink ‘View details’ will produce a detailed list of these studies, with PubMed and GEO links (where available), as well as the number of the total samples in each of the datasets. Users could then choose to go to the main BCCTBbp to mine the results published on these samples (see Figure 1).

Users of the BCCTB Sample Finder (described elsewhere, manuscript in peer-review) automatically access BCCTBbp. While assessing the availability of tissues of interest for their particular project, the Tissue Bank user will simultaneously see any literature currently available in BCCTBbp on similar sample sets (Figure 2). The BCCTBbp uses an enhanced controlled vocabulary from the BCCTB, which only has terms for the tissue types that are represented in the bank. However an internal mapping has been created to maximise the cross-querying of the two resources. For example, the Sample Finder was used to retrieve ILC samples available from BCCTB (Figure 2). Clicking on the bioinformatics details reveals 18 published studies involving this type of sample, allowing researchers to quickly identify studies similar to their research interests (Figure 2). Further mining of ILC data through BCCTBbp directly is presented in Figure 1.

### Data connectivity and interoperability

BCCTBbp is also a DAS server (8) and can be used in other resources or browsers such as Ensembl GeneView (9) using the GeneDAS protocol. Access is also possible via web services through third party software tools that have been made compatible with BioMart resources (5), such as programmatic access via BioMart APIs, R/BioConductor packages (10), Galaxy (11) and Cytoscape (12). The database is also accessible as a LinkOut resource from NCBI EntrezGene (13) and the International Cancer Genome Consortium data portal (14) (release 13).

## ANALYTICAL LAYER

The BCCTBbp additionally contains an integrated analytical tools resource for the interactive assessment of BCCTBbp datasets. This has been integrated within the web interface and facilitates tailored analysis of key datasets based on individual user interests allowing users to simply and efficiently pose questions not addressed in the original publications. This greatly expands the utility of the BCCTBbp.

**Figure 3.**
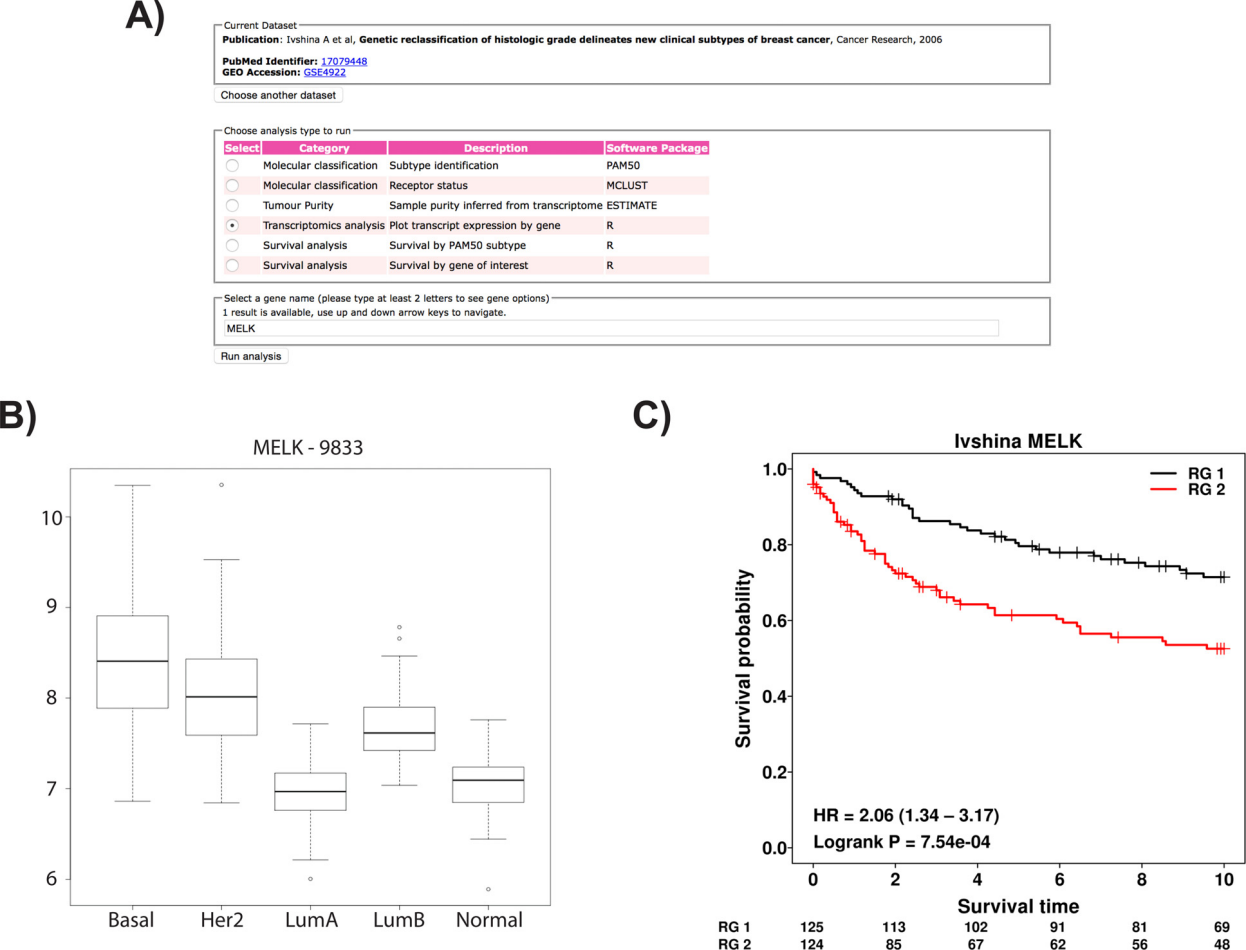
Analytical layer and integrated modules. The analytical tools can be accessed from the menu bar at http://bioinformatics.breastcancertissuebank.org/analysisTools.html. First one could choose the dataset of interest by pressing the radio-buttons to the left of the dataset title. For this example we are selecting the dataset from (**A**). Ivshina *et al.* as it has the largest sample size and contains survival information (http://bioinformatics.breastcancertissuebank.org/analysisTools.html?dset=Ivshina). (**B**) Selecting the ‘Transcriptomics Analysis’ option, and giving a gene name, MELK in this example, will result in the analysis of MELK gene (Entrez Gene ID: 9833) expression per molecular subtype (**C**) Returning to the analysis screen and selecting ‘Survival analysis by gene of interest’ for the MELK gene will produce the survival figure for two sub-groups automatically selected by the median value of MELK gene expression. Risk group assignment is presented with RG1 (black) for low expression and RG2 (red) for high expression of MELK.

Four analysis categories can be performed: Molecular Classification; Tumour Purity; Gene Expression; and Survival. Each of the included dataset samples can be classified based on the PAM50 set of markers (15) or the hormonal receptor status can be inferred from the transcriptome using the MCLUST R package (http://cran.r-project.org/web/packages/mclust/). Cancer samples frequently contain a small proportion of normal adjacent tissue that might confuse sample analysis. A method to infer sample cancer purity is implemented using ESTIMATE (16). Gene expression plots can be obtained for a gene of interest (Figure 3). Finally, for datasets that contain patient survival information, survival analysis can be performed using the ‘survival’ package in R (http://cran.r-project.org/web/packages/survival/). Two modes are available, survival based on sample sub-groups within the dataset or survival based on the expression value of a gene of interest. In the gene-based survival analysis, two classes of gene expression values (high and low) are determined based on the median expression value (Figure 3).

## ADDITIONAL FEATURES

### Data upload

To engage the wider research community, we have created provision for researchers to upload their own datasets of interest. The upload function is kept simple and requires a minimal set of information relevant to the study. For example, the user should provide information on the publication describing the dataset (e.g. title, authors and journal) as well as information on the experiment that originated the results, namely technology and biological comparison. Extra fields are also provided if the user wants to upload more details about the experiment. Finally, the user needs to upload the findings (e.g. gene/protein regulation data) in a pre-formatted excel file. Once submitted, the BCCTBbp team checks the uploaded data before inclusion in the database. Users are asked to provide an email address for contact in case of issues/questions regarding the submission.

### Documentation

A basic user guide has been provided that describes the features and functionalities of the website. The guide includes step-by-step instructions to guide users through the advanced features of the website such as performing complex queries through MartView interface or using the analysis tools. It also includes detailed instructions on accessing the portal data from external resources.

## FUTURE DIRECTIONS

BCCTBbp aims to support the Tissue Bank mission and maximise its return for the scientific community. Going forward, the portal will hold the experimental results generated on samples from the Tissue Bank. As data on the specific tissues in the bank are collated and returned to the bank, BCCTBbp will make this available to researchers thus building a valuable resource of information on the tissues in BCCTB that will save repetition of experiments and expand scientific knowledge. While focusing on increasing the data content, new graphical and analytical features will also be implemented to accommodate new data types and technologies.

New technologies for translational research are increasingly being developed to bridge the gap between clinical and research data. Developments in platforms such as TransMART (http://transmartfoundation.org/) are being closely monitored and will be assessed in future releases of BCCTBbp to move the platform from being a bioinformatics resource to providing a complete infrastructure for translational research.

While the literature and analytical components of the bioinformatics portal are undoubtedly valuable stand-alone resources, the full potential of the unique relationship between BCCTB and BCCTBbp will only become apparent in the upcoming years as the volume of valuable data about the samples available from the Tissue Bank accumulates.

## SUPPLEMENTARY DATA

Supplementary Data are available at NAR Online.
